# Phase transitions of the ionic Hubbard model on the honeycomb lattice

**DOI:** 10.1038/srep09810

**Published:** 2015-05-11

**Authors:** Heng-Fu Lin, Hai-Di Liu, Hong-Shuai Tao, Wu-Ming Liu

**Affiliations:** 1 Beijing National Laboratory for Condensed Matter Physics, Institute of Physics, Chinese Academy of Sciences, Beijing 100190, China

## Abstract

Many-body problem on the honeycomb lattice systems have been the subject of considerable experimental and theoretical interest. Here we investigate the phase transitions of the ionic Hubbard model on the honeycomb lattice with an alternate ionic potential for the half filling and hole doping cases by means of cellular dynamical mean field theory combining with continue time quantum Monte Carlo as an impurity solver. At half filling, as the increase of the interaction at a fixed ionic potential, we find the single particle gap decreases firstly, reaches a minimum at a critical interaction 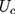, then increases upturn. At 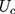, there is a band insulator to Mott insulator transition accompanying with the presence of the antiferromagnetic order. Away from half filing, the system shows three phases for the different values of hole density and interaction, paramagnetic metal, antiferromagnetic metal and ferromagnetic metal. Further, we present the staggered particle number, the double occupancy, the staggered magnetization, the uniform magnetization and the single particle spectral properties, which exhibit characteristic features for those phases.

The correlation effects in the honeycomb lattice systems have been extensively studied, which result in a number of exotic phenomena in both theory and experiment, such as the correlated electrons in the graphene^1^,^2^ and Silicene^3^,^4^,^5^, topological Mott insulator^6^ and quantum spin liquid^7^,^8^. Most of those studies are based on the standard Hubbard model, one of the most popular models in the strongly correlated system. For half-filling case, the electrons on the honeycomb lattice are described by a non-interacting massless Dirac fermion model with linear low energy dispersion relation. The system is semimetal, in which the Fermi surface are only six isolated points at the corners of the Brillouin zone. For the peculiar nature of the Fermi surface, the interaction effects can be suppressed by the low density of states in the Fermi level^9^,^10^,^11^,^12^. Away from the half-filling, the different behavior will arise in this system^13^. For example, at the 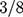 or 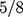 filling, the system shows many weak coupling instabilities to various ordered states, including spin density waves^14^, Pomeranchuk metal^15^, and p/d-wave superconductors^16^,^17^,^18^.

Recently, a new class of two dimensional materials 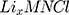 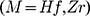 has been found^19^,^20^,^21^, which is formed on a single layer honeycomb lattice consisting of alternating “M” and “N” orbitals with a level offset. Experimental results show that 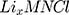 supports a unconventional superconductor^19^,^22^. The origin of superconductivity can be revealed based on the ionic-Hubbard model on the honeycomb lattice with the staggered lattice potential. The ionic Hubbard model, an extended version of the Hubbard model, was proposed to explain the neutral-ionic transition in the quasi-one-dimensional charge-transfer organic materials^23^. It has also been proposed to investigate the band insulator to Mott insulator transition, such as the one dimension system^24^,^25^ and two dimension square lattice system^26^. However, the charge dynamics with spins and the phase diagram of this model on the honeycomb lattice have not been studied. Moreover, the ionic Hubbard model on the honeycomb lattice can also be realized by cold atoms loaded in the optical lattices^27^,^28^,^29^,^30^,^31^,^32^, in which the on-site interaction, hopping amplitude, doping, and temperature can be fully controlled using Feshbach resonances, changing the lattice depth, changing the number of fermions, and varying the cooling time.

The dynamical mean field theory (DMFT)^33^ and its cluster extensions^34^,^35^ are powerful method to investigate the strongly correlated system, due to the efficient description of the quantum fluctuations. The cellular dynamical mean field theory (CDMFT)^35^ is one of the cluster extensions of DMFT, and the cluster is constructed in real lattice space. In contrast to a single site is chosen to construct the self-consistent equation in DMFT, the CDMFT picks up a cluster. This makes it is possible to include short range spatial fluctuations inside the cluster, which are important in the low dimensional systems. This method have been used to study the correlation effects on the honeycomb lattice and square lattice, such as Mott transition^36^,^37^, topological phase transition^38^ and charge order insulator transition^39^,^40^.

In this paper, we study the phase transitions of the ionic Hubbard model on the honeycomb lattice as a function of the hole doping and temperature. We adopt the CDMFT combined with the continuous time quantum Monte Carlo method (CTQMC)^41^,^42^. In order to determine the phase diagram, we calculate the staggered particle number, the double occupancy, the staggered magnetization, the uniform magnetization and the single particle spectral properties. At half filling, the system goes from a paramagnetic band insulator phase to an antiferromagnetic Mott insulator phase with the increase of the interaction. At small hole doping, the system has two phases, a paramagnetic metal for weak interaction and an antiferromangetic metal for large interaction. For finite hole doping above a critical value, the system shows three phases, a paramagnetic metal at weak interaction region, a antiferromagnetic metal at intermediate interaction region, then a ferromagnetic metal at strong interaction region.

## Results

### The strongly correlated honeycomb lattice with staggered potential

**Figure 1:**
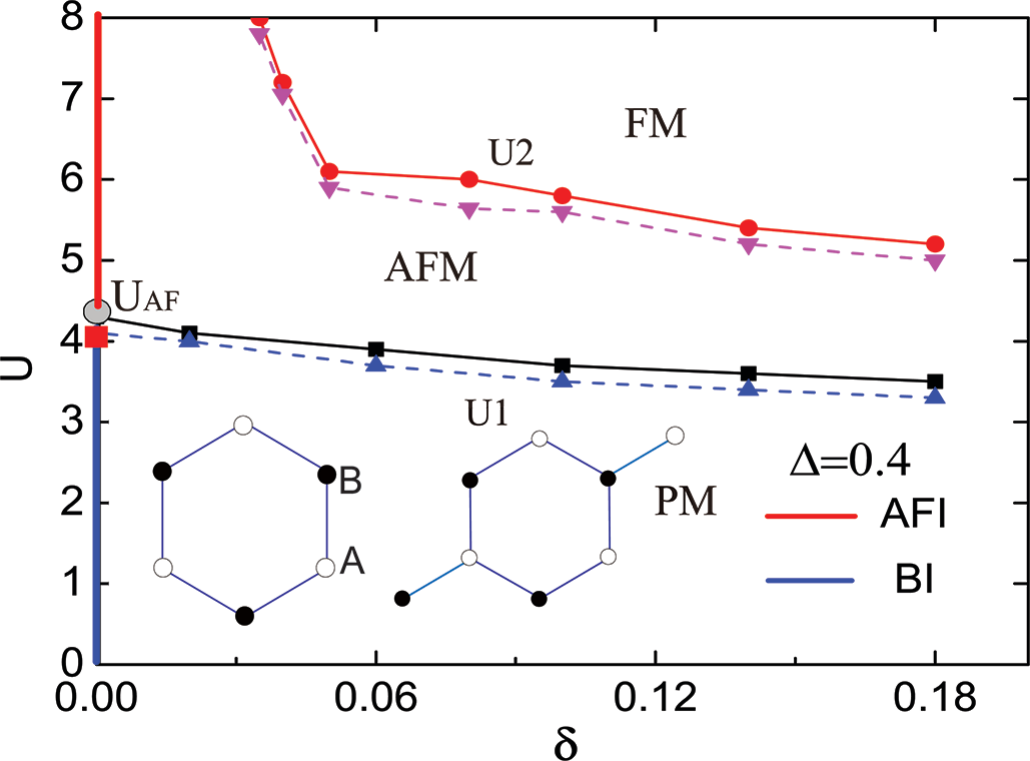
Phase diagram of the inonic Hubbard model on the honeycomb lattice. Phase diagram of the inonic Hubbard model on the honeycomb lattice at staggered potential 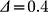 and temperature 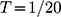. Solid lines and dash lines are the results obtained using the 6-site cluster and 8-site cluster, respectively. At half filling, the small 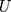 band insulator becomes an antiferromagnetic insulator at 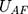. Upon doping, the system shows three phases as the alteration of the interaction strength: paramagnetic metal, antiferromagnetic metal and ferromagnetic metal. Inset: The typical clusters used within the cellular dynamical mean field theory.

We consider the ionic Hubbard model on the honeycomb lattice (see inset in Fig. 1). The system is composed of two alternating sublattices 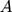 and 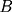. The Hamiltonian can be written as 
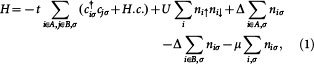
where 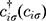 creates (destroys) an electron with spin 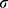 at site 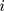. 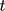 is the hopping amplitudes of fermions over nearest-neighbor sites, and we set 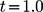 as the unit energy. 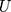 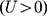 is the amplitude of the on-site repulsive interaction, and 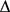 is a staggered one-body potential on the two sublattices in each unit cell, which is also called the “ionic" potential. The last term, the chemical potential 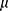 is fixed so that the average occupancy is 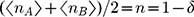, where 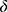 is the hole density.

We begin with the tight-binding Hamiltonian with staggered potential on the honeycomb lattice, corresponding to that the interaction 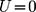 in the ionic Hubbard model. After the fourier transformation, we can get the dispersion of the free electrons, 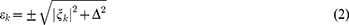
where 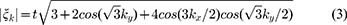
In this system, there are two bands, and the energy gap of the two bands 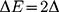. From the tight binding model analysis above, we can learn that the system can be adjusted to various phases: a semimetal when staggered potential 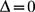 and hole doping density 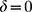, a band insulator when staggered potential 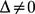 and hole doping 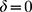, and a normal metal when staggered potential 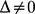 (or 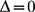) and hole doping 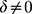. In this paper, we mainly study the correlation effects in the band insulator and hole doping band insulator.

### Phase diagram of the ionic Hubbard model

In this section, we summarize our main results of the ionic Hubbard model on the honeycomb lattice, deferring the details of how they were obtained to the following sections. The phase diagram as a function of interaction 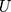 for half filling and hole doping at staggered potential 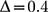 and temperature 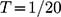 obtained from the analysis using 6-site cluster is shown in Fig. 1. The results obtained using 8-site cluster are also shown to quantitatively see the cluster-size dependence. In the noninteracting limit 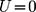, the system is band insulator and normal metal at half filling and hole doping cases, respectively. With the increase of the interaction 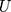, the system shows two phases for the half filling case, corresponding to the band insulator and the antiferromagnetic Mott insulator, and the two phases separate at the critical interaction 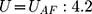. Below the critical interaction 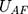, the energy gap in the band insulator are the same for both spin components and decrease as the interaction increasing. In the Mott insulator phase, the single particle energy gap are different for both spin components, such as 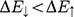. And the Mott gap increase monotonously with the increase of the interaction.

For the small hole doping case, the system goes a phase transition from paramagnetic metal to antiferromagnetic metal when changing the interaction 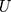. At the hole density 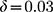, the phase transition occurs at critical interaction 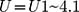. For finite hole doping above a critical value, there are three phases at different interaction, corresponding to paramagnetic metal, antiferromagnetic metal, and ferromagnetic metal. For example, at hole doping 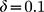, the system is paramagnetic metal below a critical interaction 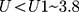, ferromagnetic metal above another critical interaction 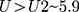, and antiferromagnetic metal between those two interaction 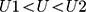.

In Fig. 1, we also present the results for the 8-site cluster. In this case, the properties of this system are qualitatively same, but the phase boundary shifts a little, such as, in 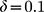, the phase transition of paramagnetic metal to antiferromagnetic metal is at 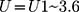 (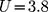 for 6-site cluster), and the antiferromagnetic metal to ferromagnetic metal is at 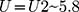 (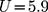 for 6-site cluster). We describe below in details of the spectral and magnetic properties that lead to this diagram.

### Local quantities and spectral properties for the half filling case 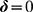


In this section, we firstly try to understand the correlation effects on the band insulator on the honeycomb lattice. We concentrate on the half-filling case 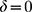 for different values of the staggered potential 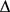 with the average occupancy 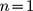. In the noninteracting limit, the system prefers a band insulator phase, in which most of the electrons stay on a sublattice with lower potential, resulting in zero density of states in the Fermi surface. When the local interaction is turned on, the band insulator competes with the Mott insulator with one electron per lattice site. In Fig. 1, the phase diagram give us the results of the band insulator to Mott insulator at 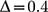 and 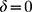. Here we will give more detailed description on the results for different values of staggered potential 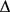.

In order to examine how the system evolves with the variation of the local interaction, we firstly calculate the four local quantities: staggered charge density 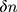, double occupancy 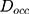, staggered magnetization 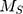, and uniform magnetization 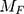. The staggered charge density and double occupancy are related with the charge fluctuation, the staggered magnetization and the uniform magnetization give us the information about the spin fluctuation. The staggered charge density is defined by the difference between the particle number densities at two sublattices, 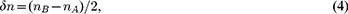
where the sublattice number densities can be calculated as 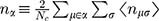 for 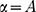 and 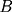, 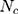 is the site numbers of the cluster. We also calculate the double occupancy defined by 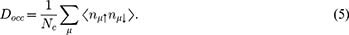


The staggered magnetization and uniform magnetization are defined as 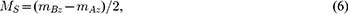
and 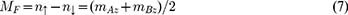
respectively, where the sublattice magnetization is calculated as 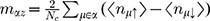 for 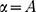 and 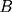.

**Figure 2:**
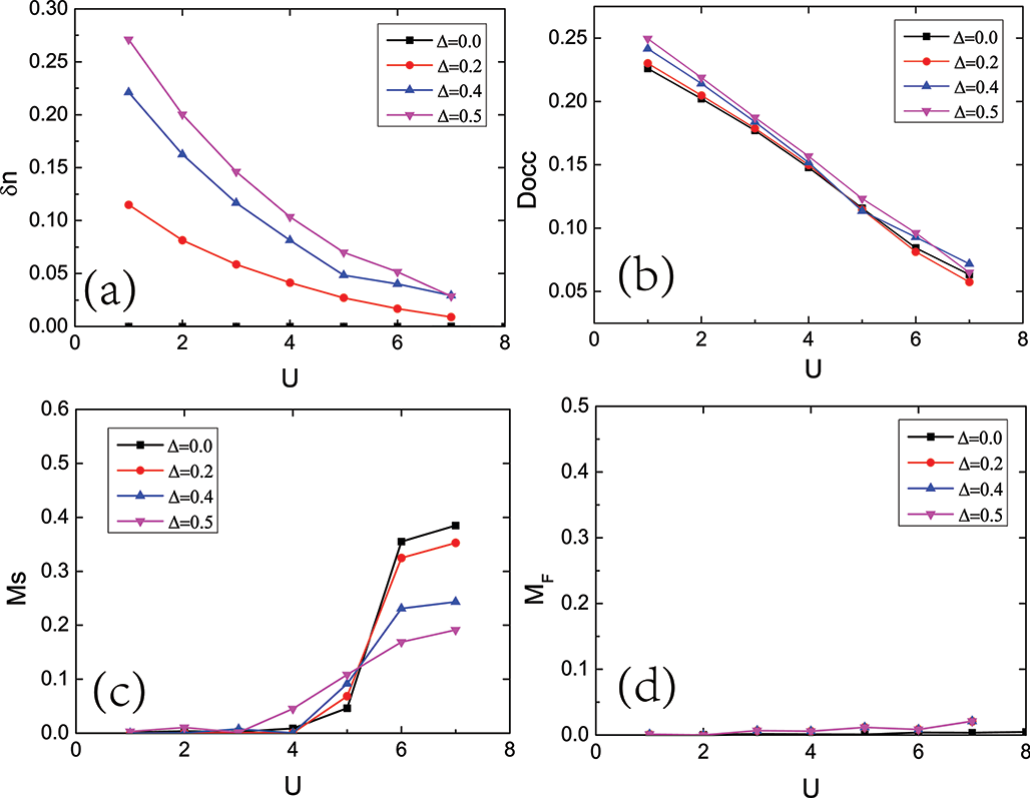
Four local quantities as a function of interaction 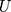 for the half filling case. Four local quantities as a function of interaction 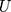 for temperature 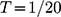 and various 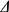 values. (a) Staggered charge density 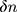. (b) Double occupancy 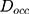. (c) Staggered magnetization 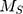. (d) Uniform magnetization 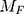.

The results for the staggered charge density 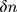 and the double occupancy 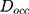 as a function of interaction 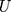 for temperature 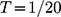 are shown in Figs. 2(a) and 2(b). Due to the staggered on-site potential, 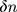 is always nonzero, even thought the Hubbard 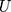 tries to suppress it. 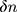 decreases monotonically as a function of 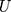, and shows no discontinuity at 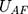. In the weak interaction region, the electrons prefer to gather on the lower potential sublattice 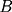. The system experiences an imbalance between the two sublattice, resulting in higher double occupancy, compared with the Hubbard model when 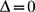 and a nonzero staggered charge density. Such tendencies become stronger as 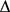 grows. In the ionic limit 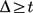, it is energetically favorable that all the electrons are in the sublattice 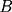, producing unity of the staggered charge. As 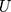 increasing, the energy cost of two electrons to stay in the same site becomes large, both the double occupancy and the staggered charge density decrease monotonically with the imbalance between the two sublattices become weaker. In the strong coupling limit, the staggered charge density is close to 0.

In Figs. 2(c) and 2(d) we plot the staggered magnetization 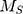 and uniform magnetization 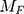 as a function of interaction 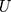 for temperature 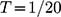 respectively. For a given 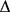, there exists a threshold value 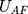 at which the staggered magnetization turns on with a jump. Both the value of the 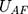 and the amplitude of the jump in 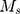 are decreasing functions of 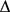. In the half filling case 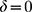, the uniform magnetization 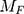 is almost zero, independent of the staggered potential 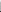 and interaction strength 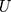.

**Figure 3:**
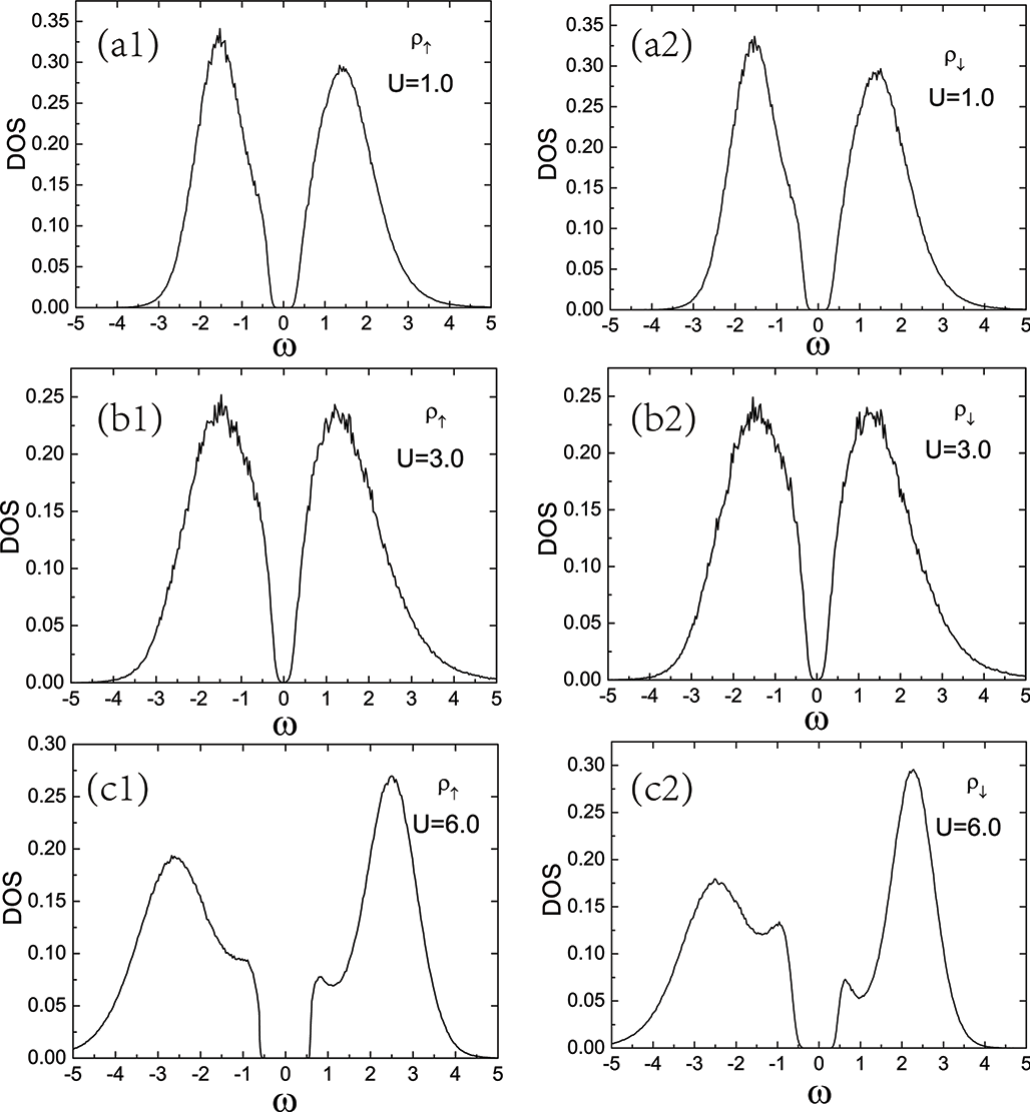
Spin-resolved single particle density of states 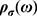 for the half filling case. Spin-resolved single particle density of states 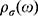 as a function of 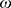 for 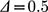. (a1) and (a2) Band insulator for U = 1.0 with large band gap and spin symmetry of the 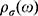 . (b1) and (b2) Band insulator for U = 3.0 with small band gap and spin symmetry of the 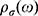, (c1)and (c2) Mott insulator for U = 6.0 with large Mott gap and without spin symmetry of the 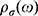.

**Figure 4:**
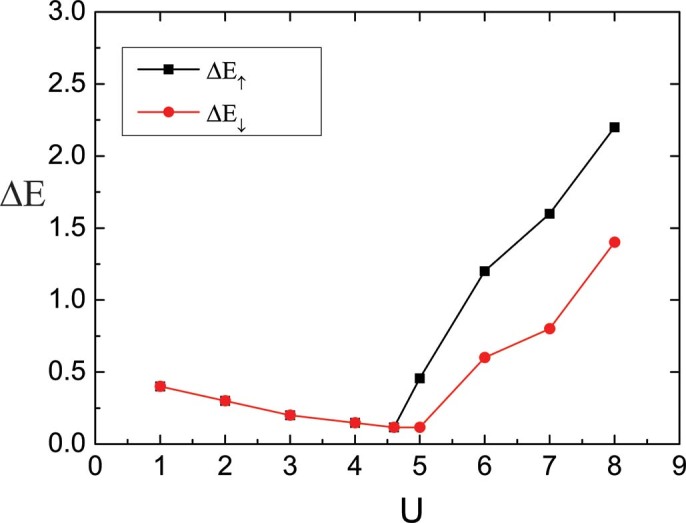
Spectral gaps of the two spin components 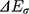. Spectral gaps of the two spin components 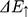 and 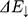 as a function of the interaction strength 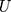 for 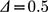 at temperature 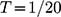. With the increase of 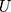 the spectral gap decreases for weak interaction while it grows larger in the region of strong interactions.

The local density of states provide more detailed information on the single particle properties. The spin-resolved single particle density of states are computed as follows 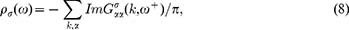
where 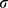 is the spin, 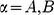, and 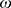 is measured from the chemical potential 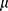. The density of states are derived from the imaginary time Green’s function 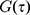 using maximum entropy method^43^. The local density of states are shown in Fig. 3 for several values of 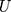 at staggered potential 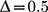. For a quantitative analysis of the gap around a Fermi level, we investigate the spectral gap 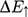 and 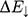 for both spin components which are defined as the energy difference between the highest filled and lowest empty levels in the local density of states. Fig. 4 shows the spectral gap as a function of the interaction strength 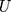 for 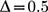 at temperature 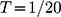. In the noninteracting system 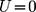, the local density of states can be computed analytically and is composed of two bands which are separated by a band gap 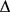 due to the staggered potential. For weak interaction, the local density of states are the same for both spin components. However the band gap around a Fermi level decreases monotonically with the increase of interaction in this region. For example, the density of states for two spin components at interaction 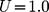 and 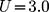 are shown in Figs. 3(a1)(a2) and Figs. 3(b1)(b2) respectively, the band gap at 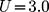 is smaller than 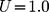. The local density of states displays a minimum spectral gap at a critical value of interaction 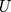, 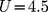 for up-spin component and 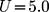 for down-spin component. When 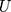 above the critical value, the gap between the two bands in turn is enlarged, and the system goes to the Mott insulator phase. In this region, the local density of states for up-spin component and down-spin component show different behaviors (Figs. 3(c1)(c2). The Mott gap of the up-spin component is bigger than the down-spin component.

### Local quantities and spectral properties for the hole doping case 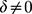


In this section, we now turn to study the phase transitions of the hole doping (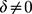) band insulator as a function of the interaction on the honeycomb lattice. The average occupancy 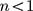, resulting in finite density of states in the Fermi level. The system is a metal, and the low energy physics can be described by the Fermi liquid theory. When the interaction is turned on, there are many instabilities for the Fermi liquids, which is an enduring theme research in condensed matter physics. When the staggered potential 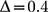 and the temperature 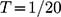, the phase diagram for different values of hole doping 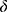 is displayed in Fig. 1. For small hole doping 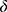, the system goes a phase transition from paramagnetic metal to antiferromagnetic metal at a critical interaction 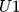. At finite hole doping 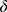 above a critical value, the system has three phases as the interaction 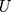 varying, paramagnetic metal when 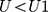, antiferromagnetic metal when 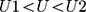, and ferromagnetic metal when 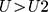. Now we discuss how we use the single particle spectral and other local qualities to determine the phase boundaries 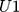 and 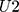.

**Figure 5:**
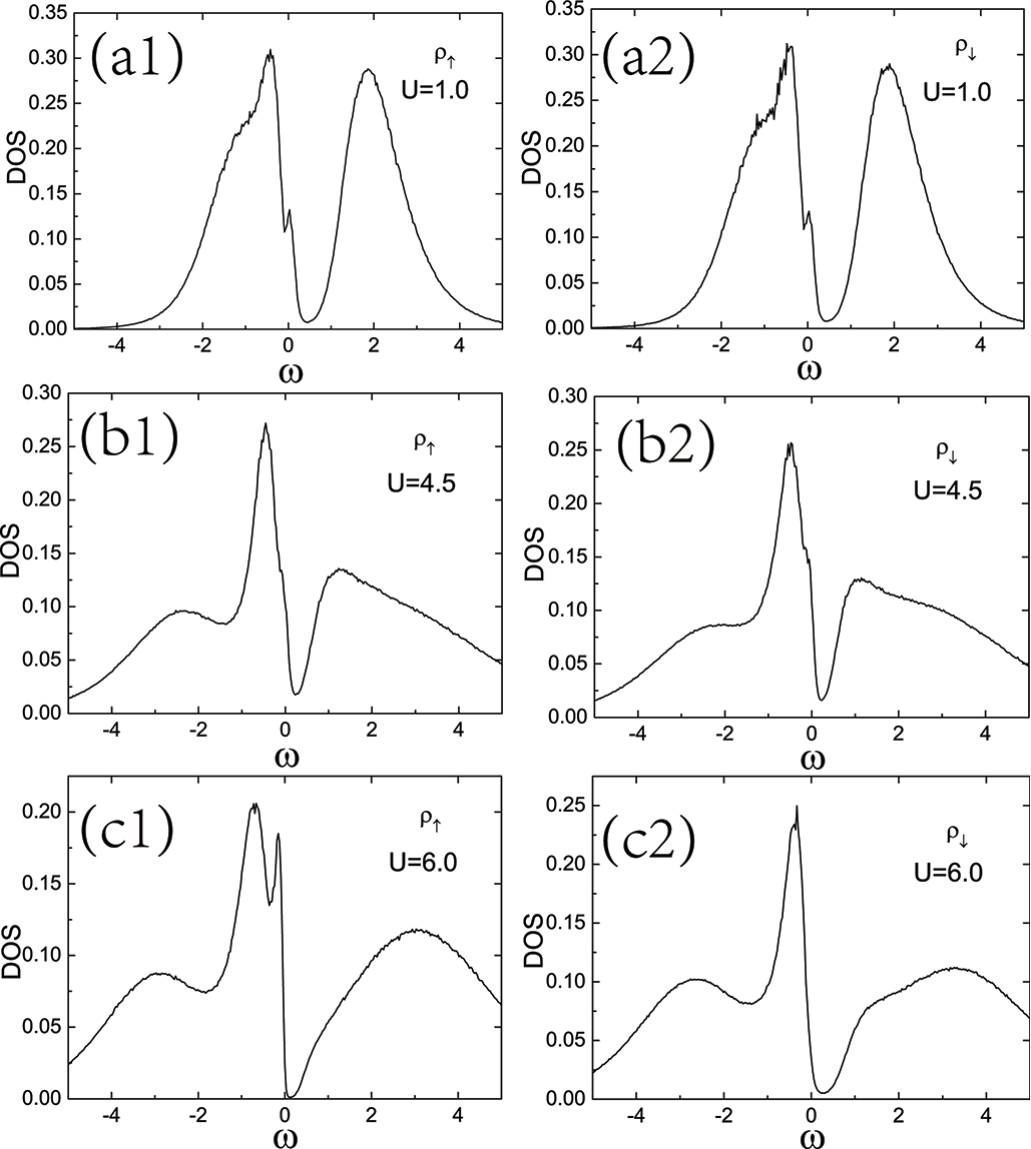
Spin-resolved single particle density of states 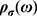 for the hole doping case. Spin-resolved single particle density of states 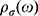 as a function of 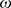 for 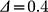 at hole doping 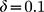 and temperature 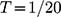. (a1) and (a2) Paramagnetic metal for U = 1.0 with spin symmetry of the 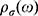. (b1) and (b2) Antiferromagnetic metal for U = 4.5 with spin symmetry of the 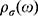, (c1)and (c2) Ferromagnetic metal for U = 6.0 without spin symmetry of the 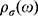.

Firstly, let us study the single particle density of states as a function of interaction for various hole doping. Fig. 5 shows the single particle density of states 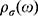 for both of the spin components at hole doping 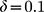 and staggered potential 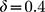
_$_. The single particle density of states 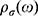 are obtained from Eq.(8). With the increase of the interaction, the spectral weight is continuously transferred to the higher energy states, the chemical potential lies inside the lower band for both spin components all the time. When 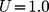, in the paramagnetic metal region, the density of states for both spins are the same, and there are two the spectral peaks above and below the Fermi level (Figs. 5(a1) and 5(a2)). When 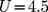, corresponding to the antiferromagnetic metal, the antiferromagnetic order sets in, making the density of states and gaps a little different for the two spin components (Figs. 5(b1) and 5(b2)). When 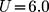, in the ferromangnetic metal region, the density of states for the two spin components are renormalized much and very different (Figs. 5(c1) and 5(c2)). In both the antiferromagnetic metal and ferromagnetic metal, one of the spectral peaks above the Fermi level is suppressed.

**Figure 6:**
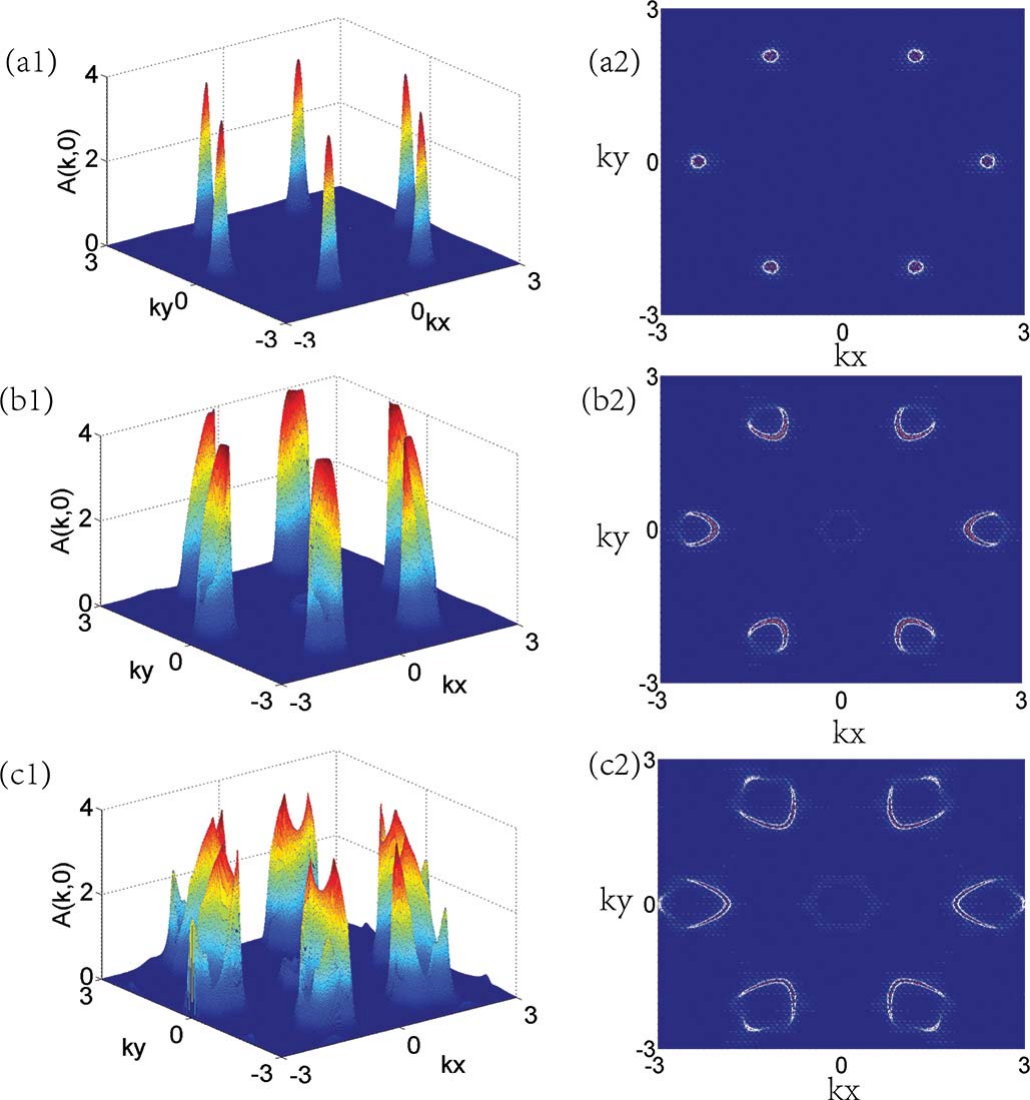
The distribution of low energy spectral weight 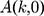 in 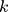 space. The distribution of low energy spectral weight in 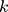 space 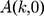 at temperature 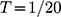 for different interactions 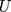. (a1) and (a2) 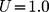, (b1) and (b2) 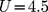, (c1) and (c2) 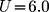. The right panels are color plots to see the Fermi surface and left panels are three dimensional plots to see the variation of 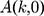.

Besides the changes of the local density of states, the interaction will influence the momentum-resolved spectral density in the Fermi level 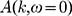 very much. The 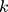-resolved spectral weight can be defined as 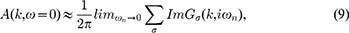
which are the maxima of the spectral weight at zero temperature as a function of 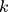. In Fig. 6 we present 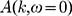 for the three different phases at hole doping 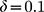 and staggered potential 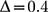. In the hole doping case, the Fermi surface 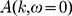 are six rings in the 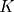 and 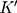 points located at the corners of the hexagon. When the interaction is small 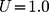, corresponding to paramagnetic metal, the Fermi surface is only weakly renormalized compared to the case of the interaction 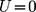 (Figs. 6(a1)(a2)). In the intermediate interaction region 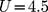, corresponding to antiferromagnetic metal, the distribution of quasi-particle spectral near the 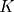 and 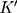 points became anisotropic in different directions in each corners (Figs. 6(b1)(b2)). In the large interaction region 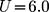, corresponding to ferromagnetic metal, the Fermi surface are strongly renormalized at each corners, the peak of 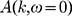 are broadened (Figs. 6(c1)(c2)).

**Figure 7:**
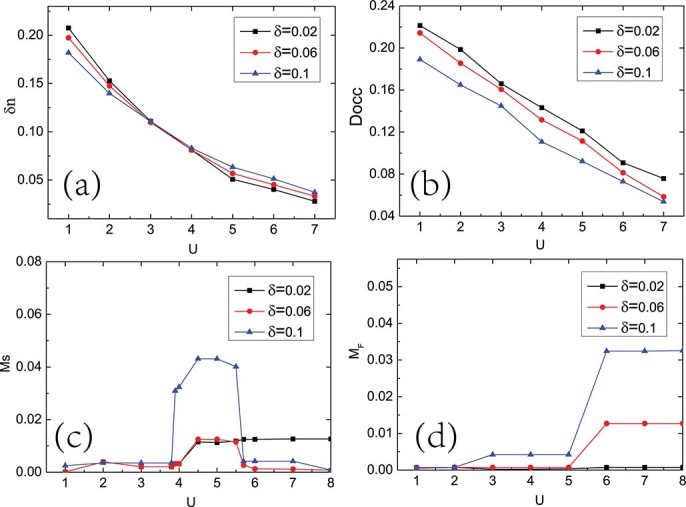
Four local quantities as a function of interaction 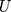 for the hole doping case. Four local quantities as a function of interaction 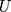 for temperature 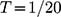 for various doping 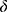 values. (a) Staggered charge density 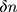. (b) Double occupancy 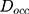. (c) Staggered magnetization 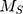. (d) Uniform magnetization 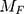.

In addition to the spectral properties, we also calculate four local quantities: the staggered charge density 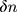, the double occupancy 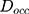, the staggered magnetization 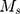, and the uniform magnetization 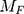 obtained from Eqs.(4), (5), (6) and (7), respectively. Figs. 7(a)(b) show the staggered charge density 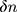 and the double occupancy 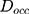 as a function of 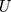 for hole doping 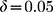, 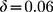 and 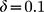 at staggered potential 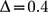. Although the system is metallic all the times, with the increase of the interaction, both of the two quantities decrease monotonically. Figs. 7(c)(d) show the staggered magnetization 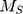 and the uniform magnetization 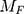 as a function of interaction for hole doping 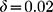, 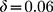 and 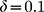 at staggered potential 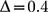. For small hole doping, such as 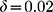, the system shows a phase transition from paramagnetic metal to antiferromagnetic metal in which the staggered magnetization 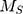 turns on a finite value at 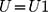, and the uniform magnetization 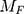 is zero all the time. When the hole doping above a critical value, such as 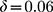 and 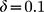, the magnetic properties shows dramatic changes at the phase boundaries 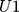 and 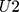. For small 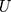, 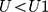, the magnetic order is not favored. For intermediate 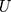, 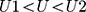, there is a nonzero staggered magnetization 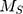. When the 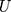 is large, 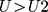, the nonzero staggered magnetization 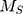 is suppressed, the uniform magnetization 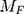 becomes a nonezero value. Both of the staggered magnetization 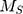 and the uniform magnetization 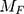 increase with the increase of the hole doping 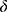.

## Discussion

In this work, we have investigated the effect of on-site interaction and staggered ionic potential in a band insulator and doped band insulator on the honeycomb lattice based on the ionic Hubbard model. By means of the cellular dynamical mean field theory combing with continue time Monte Carlo method, we construct a phase diagram as a function of interaction and hole doping. At half filling, although the single particle spectral functions always posses a energy gap, the system shows a band insulator to Mott insulator transition at a critical interaction 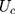, with the single particle gap decreases firstly, reaches a minimum at a critical interaction 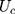, then increases upturn, and the antiferromagnetic order gives a finite value above 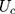. Away from half filing, many metallic phases with magnetic order are found, in order to exhibit characteristic features of the phases, the behavior of the staggered particle number, the double occupancy, the staggered magnetization, the uniform magnetization and the single particle spectral properties have bend studied. At small hole doping, the system goes a phase transition from a paramagnetic metal to an antiferromangetic metal with the increase of the interaction. For finite hole doping above a critical value, the system shows three phases, a paramagnetic metal at weak interaction region, a antiferromagnetic metal at intermediate interaction region, then a ferromagnetic metal at strong interaction region.

We get itinerant metals with spin density wave state which are an interesting class of materials where electrons show spin polarization or staggered spin polarization behavior. They have applications in spintronics as they can generate spin-polarized currents^44^,^45^,^46^. And the materials with the honeycomb lattice structure are very common, such as single layer graphene, silicene considered as the silicon-based counterpart of graphene, and monolayer molybdenum disulfide (ML-MDS), MoS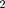, which play a vital role in nanoelectronics and nanospintronics. We hope that our study will motivate a research on along those lines and open up new possibilities in the area of spintronics. Moreover, with the development of the cold atom experiment, the honeycomb lattice have been simulated^29^,^32^,^47^,^48^, which can give us a platform to simulate and detect the phase transitions by loading ultracold atoms on the honeycomb optical lattices.

## Methods

In order to study the ionic Hubbard model in honeycomb lattice which describes the correlation effects on the band insulator and the hole doped band insulator, the Cellular dynamical mean field theory are employed. The Cellular dynamical mean field theory is an extension of dynamical mean field theory, which is able to partially cure dynamical mean field theory’s spatial limitations. We replace the single site impurity by a cluster of impurities embedded in a self-consistent bath. The cluster-impurity problem embedded in a bath of free fermions can be written in a quadratic form, 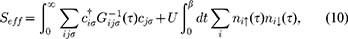
where i and j are the coordinates inside the cluster-impurity, and the 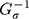 is the Weiss field. The effective medium 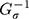 is computed via the Dyson equation, 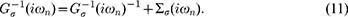


Within cellular dynamical mean field theory, the interacting lattice Green’s function in the cluster site basis is given by, 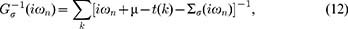
where 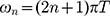 are Matsubara frequencies, 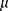 is the chemical potential and 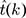 is the Fourier-transformed hopping matrix for the super lattice. In our analysis, the 6- and 8-site clusters in the inset of Fig. 1 are used to set up the cluster Hamiltonian. For the 6-site cluster case, the hopping matrix 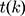 of the cluster can be written as follows (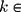 the reduced Brillouin-zone), 
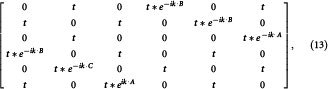
where 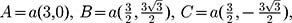 are the nearest-neighbor super-lattice vectors, 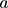 is the lattice constant. In each iteration, in order to solve the effective cluster model and to calculate 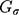, we use the weak coupling interaction expansion continuous time quantum Monte Carlo method.

The CDMFT iteration procedure is summary as follows. Given a cluster self-energy 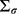, we can compute 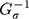 via Eq.(5)(6), then solve the effective cluster model and to calculate a new 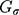. Then us Eq.(5) again, we can get a new cluster self-energy 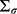. Repeat the procedure until the results are convergence.

The weak coupling interaction expansion continuous time quantum Monte Carlo method is efficient method to treat the impurity model. The method employs same tricks, which used to derived Feynman perturbation theory, to stochastically generate the partition function 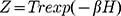. In the interaction picture, 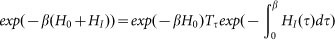, where 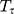 is the time-ordering operator. The expansion of the partition function in power of 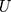 reads 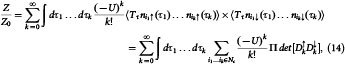
where 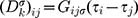 and 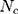 is the number of the sites of the cluster. The observable expectation value 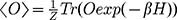 can be sampled during the Monte Carlo update. For example, the Green’s function 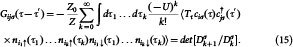


## References

[b1] Neto A. H. C., Guinea F., Peres N. M. R., Novoselov K. S. & Geim A. K. The electronic properties of graphene. Rev. Mod. Phys. 81, 109 (2006). 10.1103/PhysRevLett.97.266801 17280447

[b2] Kotov V. N., Uchoa B., Pereira V. M., Guinea F., & Neto A. H. C. Electron-Electron Interactions in Graphene: Current Status and Perspectives. Rev. Mod. Phys. 84, 1067 (2012).

[b3] GuzmanVerri G. G. & Lew Yan Voon L. C. Electronic structure of silicon-based nanostructures. Phys. Rev. B 76, 075131 (2007).

[b4] Vogt P. *et al.* Silicene: Compelling Experimental Evidence for Graphenelike Two-Dimensional Silicon. Phys. Rev. Lett. 108, 155501 (2012). 22587265 10.1103/PhysRevLett.108.155501

[b5] Fleurence A. *et al.* Experimental Evidence for Epitaxial Silicene on Diboride Thin Films. Phys. Rev. Lett. 108, 245501 (2012). 23004288 10.1103/PhysRevLett.108.245501

[b6] Raghu S., Qi X. L. Honerkamp C. & Zhang S. C. Topological Mott Insulators. Phys. Rev. Lett. 100, 156401 (2008). 18518132 10.1103/PhysRevLett.100.156401

[b7] Meng Z. Y., Lang T. C., Wessel S., Assaad F. F. & Muramatsu A. Quantum spin liquid emerging in two-dimensional correlated Dirac fermions. Nature 464, 847 (2010). 20376143 10.1038/nature08942

[b8] Assaad F. F. & Herbut I. F. Pinning the Order: The Nature of Quantum Criticality in the Hubbard Model on Honeycomb Lattice. Phys. Rev. X 3, 031010 (2013).

[b9] Sorella S. & Tosatti E. Semi-Metal-Insulator Transition of the Hubbard Model in the Honeycomb Lattice. Europhys. Lett. 19, 699 (1992).

[b10] Paiva T., Scalettar R. T., Zheng W., Singh R. R. P. & Oitmaa J. Ground-state and finite-temperature signatures of quantum phase transitions in the half-filled Hubbard model on a honeycomb lattice. Phys. Rev. B 72, 085123 (2005).

[b11] Herbut I. F. Interactions and Phase Transitions on Graphenes Honeycomb Lattice. Phys. Rev. Lett. 97, 146401 (2006). 17155272 10.1103/PhysRevLett.97.146401

[b12] Herbut I. F., Jurii V. & Vafek O. Relativistic Mott criticality in graphene. Phys. Rev. B 80, 075432 (2009).

[b13] McChesney, J. L. . Extended van Hove Singularity and Superconducting Instability in Doped Graphene. *Phys. Rev. Lett.* **104**, 136803 (2010). 10.1103/PhysRevLett.104.136803 20481902

[b14] Makogon D., van Gelderen R., Roldan R. & Smith C. M. Spin-density-wave instability in graphene doped near the van Hove singularity. Phys. Rev. B 84, 125404 (2011).

[b15] Valenzuela B. & Vozmediano M. A. H. Pomeranchuk instability in doped graphene. New J. Phys. 10, 113009 (2008).

[b16] Nandkishore R., Levitov L. & Chubukov A. Chiral superconductivity from repulsive interactions in doped graphene. Nature Phys. 8, 158 (2012).

[b17] Gonzalez J. Kohn-Luttinger superconductivity in graphene. Phys. Rev. B 78, 205431 (2008). 10.1103/PhysRevLett.122.026801 30720323

[b18] Kiesel M. *et al.* Competing many-body instabilities and unconventional superconductivity in graphene. Phys. Rev. B 86, 020507 (2012).

[b19] Yamanaka S., Hotehama K. & Kawaji H. Superconductivity at 25.5 K in electron-doped layered hafnium nitride. Nature 392, 580 (1998).

[b20] Hase I. & Nishihara Y. Electronic structure of superconducting layered zirconium and hafnium nitride. Phys. Rev. B 60, 1573 (1999).

[b21] Felser C. & Seshadri R. Electronic structures and instabilities of ZrNCl and HfNCl: implications for superconductivity in the doped compounds. J. Mater. Chem. 9, 459 (1999).

[b22] Taguchi Y., Hisakabe M. & Iwasa Y. Specific Heat Measurement of the Layered Nitride Superconductor LixZrNCl. Phys. Rev. Lett. 94, 217002 (2005). 16090340 10.1103/PhysRevLett.94.217002

[b23] Nagaosa N. Theory of Neutral-Ionic Transition in Organic Crystals. III. Effect of the Electron-Lattice Interaction. J. Phys. Soc. Jpn. 55, 2754 (1986).

[b24] Batista C. D. & Aligia A. A. Exact Bond Ordered Ground State for the Transition between the Band and the Mott Insulator. Phys. Rev. Lett. 92, 246405 (2004). 15245117 10.1103/PhysRevLett.92.246405

[b25] Tincani L., Noack R. M. & Baeriswyl D. Critical properties of the band-insulator-to-Mott-insulator transition in the strong-coupling limit of the ionic Hubbard model. Phys. Rev. B 79, 165109 (2009).

[b26] Paris N., Bouadim K., Hebert F., Batrouni G. G. & Scalettar R. T. Quantum Monte Carlo Study of an Interaction-Driven Band-Insulator to Metal Transition. Phys. Rev. Lett. 98, 046403 (2007). 17358793 10.1103/PhysRevLett.98.046403

[b27] Jaksch D., Bruder C., Cirac J. I., Gardiner C. W. & Zoller P. Cold Bosonic Atoms in Optical Lattices. Phys. Rev. Lett. 81, 3108 (1998).

[b28] Hofstetter W., Cirac J. I., Zoller P., Demler E. & Lukin M. D. High-Temperature Superfluidity of Fermionic Atoms in Optical Lattices. Phys. Rev. Lett. 89, 220407 (2002). 12485058 10.1103/PhysRevLett.89.220407

[b29] Greiner M., Mandel O., Esslinger T., Hansch T. W. & Bloch I. Quantum Phase Transition from a Superfluid to a Mott Insulator in a Gas of Ultracold Atoms. Nature 415, 39 (2002). 11780110 10.1038/415039a

[b30] Duan L. M., Demler E. & Lukin M. D. Controlling Spin Exchange Interactions of Ultracold Atoms in Optical Lattices. Phys. Rev. Lett. 91, 090402 (2003). 14525163 10.1103/PhysRevLett.91.090402

[b31] Soltan-Panahi P. *et al.* Multi-Component Quantum Gases in Spin-Dependent Hexagonal Lattices. Nature Phys. 7, 434 (2011).

[b32] Gemelke N., Zhang X., Hung C.-L. & Chin C. In Situ Observation of Incompressible Mott-Insulating Domains in Ultracold Atomic Gases. Nature 460, 995 (2009). 19693080 10.1038/nature08244

[b33] Georges A., Kotliar G., Krauth W. & Rozenberg M. J. Dynamical mean field theory of strongly correlated fermion systems and the limit of infinite dimensions. Rev. Mod. Phys. 68, 13 (1996).

[b34] Maier T. Jarrell M. Pruschke T. & Hettler M. H. Quantum cluster theories. Rev. Mod. Phys. 77, 1027 (2005).

[b35] Kotliar G., Savrasov S. Y., Pálsson G. & Biroli G. Cellular dynamical mean field approach to strongly correlated Systems. Phys. Rev. Lett. 87, 186401 (2001).

[b36] Liebsch A. Correlated Dirac fermions on the honeycomb lattice studied within cluster dynamical mean field theory. Phys. Rev. B 83, 035113 (2011).

[b37] Parcollet O., Biroli G., & Kotliar G. Cluster dynamical mean field analysis of Mott transition. Phys. Rev. Lett. 92, 226402 (2004). 15245242 10.1103/PhysRevLett.92.226402

[b38] Wu W., Rachel S., Liu W. M. & Le Hur K. Quantum spin Hall insulators with interactions and lattice anisotropy. Phys. Rev. B 85, 205102 (2012).

[b39] Wu W. & Tremblay A. M. S. Phase diagram and Fermi liquid properties of the extended Hubbard model on the honeycomb lattice. Phys. Rev. B 89, 205128 (2014).

[b40] Merino J. Nonlocal coulomb correlations in metals close to a charge order insulator transition. Phys. Rev. Lett. 99, 036404 (2007). 17678302 10.1103/PhysRevLett.99.036404

[b41] Rubtsov A. N., Savkin V. V. & Lichtenstein A. I. Continuous-time quantum Monte Carlo method for fermions. Phys. Rev. B 72, 035122 (2005). 10.1103/PhysRevLett.94.026402 15698201

[b42] Gull, E., . Continuous-time Monte Carlo methods for quantum impurity models. *Rev. Mod. Phys.* **83**, 349 (2011).

[b43] Jarrell M. & Gubernatis J. E. Bayesian inference and the analytic continuation of imaginary-time quantum Monte Carlo data. Phys. Rep. 269, 133 (1996).

[b44] Pickett W. E., & Moodera J. S. Half metallic magnets. Phys. Today 54, 39 (2001).

[b45] Katnelson M. I., Irkhin V. Y., Chioncel L. & de Groot R. A. Half-metallic ferromagnets: From band structure to many-body effects. Rev. Mod. Phys. 80, 315 (2008).

[b46] Katnelson M. I., Irkhin V. Y., Chioncel L. & de Groot R. A. Half-Metallic Alloys: Fundamentals and Applications, Lecture Notes in Physics, edited by I. Galanakis, and P. H. Dederichs (Springer, New York. 2005).

[b47] Bloch I., Dalibard J. & Zwerger W. Many-body physics with ultracold gases. Rev. Mod. Phys. 80, 885 (2008).

[b48] Kechedzhi K., Falko,. Vladimir I., McCann E. & Altshuler B. L. Influence of Trigonal Warping on Interference Effects in Bilayer Graphene. Phys. Rev. Lett. 98, 176806 (2007).

